# Unprotected Left Main Percutaneous Coronary Intervention in Acute Coronary Syndromes with Extracorporeal Life Support Backup

**DOI:** 10.1155/2015/435878

**Published:** 2015-02-25

**Authors:** Dawid L. Staudacher, Oliver Langner, Paul Biever, Christoph Benk, Manfred Zehender, Christoph Bode, Tobias Wengenmayer

**Affiliations:** ^1^Department of Cardiology and Angiology, Heart Center Freiburg University, Hugstetterstrasse 55, 79106 Freiburg im Breisgau, Germany; ^2^Department of Cardiovascular Surgery, Heart Center Freiburg University, Hugstetterstrasse 55, 79106 Freiburg im Breisgau, Germany

## Abstract

*Background*. Left main PCI is superior to coronary bypass surgery in selected patients. Registry data, however, suggest significant early adverse event rates associated with unprotected left main PCI. We aimed to evaluate safety of an extracorporeal life support (ECLS) as backup system during PCI.* Methods*. We report a registry study of 16 high-risk patients presenting with acute coronary syndromes undergoing unprotected left main PCI with an ECLS backup.* Results*. Seven patients (43.8%) presented with an acute myocardial infarction while 9 patients (56.3%) had unstable angina. Unprotected left main PCI could be successfully performed in all 16 patients. Mortality or thromboembolic event rates were zero within the index hospital stay. General anesthesia was necessary only in 5 patients (31.3%). Access site bleeding requiring transfusion was encountered in 4 patients (25.0%). Three patients (18.8%) developed access site complications requiring surgical intervention. All patients were ECLS-free after 96 hours. *Conclusions*. Unprotected left main PCI could be safely and effectively performed after ECLS implantation as backup in acute coronary syndromes in our patient collectively. Vascular access site complications however need to be considered when applying ECLS as backup system.

## 1. Introduction

Finding the optimal treatment strategy for patients with significant left main coronary artery (LMCA) is challenging. For patients presenting with left main stenosis, coronary bypass graft (CABG) is superior to medical therapy [1, 2]. No data from randomized controlled trials exist comparing PCI with medical therapy [1]. Mortality rates after PCI and CABG for LMCA intervention appear to be comparable [2]. Need for target-vessel revascularization is higher after stenting and stroke incidence is higher after CABG [1]. According to the SYNTAX trial, mortality 3 and 5 years after LMCA intervention favored LMCA PCI in the low to intermediate SYNTAX score subgroup (mortality 3.7% after PCI versus 9.1% after CABG) while favoring CABG in patients with a high SYNTAX score (mortality 13.4% after PCI versus 7.6% after CABG [3–5]). Current guidelines [1] recommend a “heart team approach” for choosing the revascularization strategy for each individual patient while recommending immediate PCI for all STEMI patients with LMCA stenosis (especially those with LMCA culprit lesion and low TIMI flow and for those who are hemodynamically unstable).

According to a small case series, LMCA PCI can be performed without cardiosurgical backup [6]. Registry data, however, suggest significant early adverse event rates after LMCA PCI (19.2% mortality, 7.5% AMI, and 1.7% stroke) [7]. Routinely implanted IABP or the left ventricular assist devices like Impella or TandemHeart for high-risk PCI showed heterogeneous results [8–14]. Therefore the optimal backup system for left main PTCA in patients with high risk of hemodynamic instability during PCI is still to be determined.

Extracorporeal life support (ECLS) has been routinely used in the catheterization laboratory in patients with cardiogenic shock [15] and for patients with cardiac arrest without return of spontaneous circulation [16]. It is a modification of the cardiopulmonary bypass circuit routinely used in cardiac surgery. Specifically, the ECLS drains blood through a cannula inserted in the vena femoralis and returns oxygenated and decarboxylated blood through a cannula inserted in the arteria femoralis. A blood flow up to 5 L/min can be reached, which is sufficient even in complete cardiac arrest [15, 16]. There are several case reports and smaller case series on ECLS backup for left main PCI [17–20].

Some publications employ venoarterial extracorporeal membrane oxygenation (ECMO) synonymous with extracorporeal life support (ECLS). Since membrane oxygenation is not the indication for implantation of a venoarterial backup during PCI, ECLS (term coined by ELSO, the Extracorporeal Life Support Organization) will be used.

## 2. Methods

### 2.1. Patient Selection

We report data on all 16 patients undergoing unprotected left main PCI with ECLS backup at the Heart Center Freiburg University between March 2011 and December 2012 (patient characteristics are given in Table 1).

Within this time period, a total of 54 patients underwent LMCA PCI at our center. Of those, 13 were CABG protected and 41 underwent unprotected LMCA PCI. In the 16 patients (39%) reported in this register, the LMCA PCI was considered very high risk and an ECLS was implanted prior to the coronary intervention. Specifically, 6 patients presented in cardiogenic shock and had an emergency PCI after ECLS implantation. All the other 35 patients undergoing unprotected LMCA PCI were discussed by our heart team and PCI was considered the superior strategy over bypass surgery or medical therapy for each individual patient. Reasons for the interventional approach were high surgical risk, patient comorbidity, previous thoracic surgery, explicit patient wish, and, in one case, an active Takayasu arteritis. In 10 out of 35 patients, PCI was considered very high risk by the interventional cardiologist or the heart team and an ECLS was implanted prior to LMCA PCI.

### 2.2. ECLS Implantation

In case of an expected high risk of hemodynamic instability during PCI, implantation of an ECLS was performed prior to PCI. In patients presenting with acute myocardial infarction in cardiogenic shock and left main coronary artery culprit lesion, ECLS was implanted prior to PCI in the catheterization laboratory.

Two commercially available ECLS systems (CADIOHELP by MAQUET Medical and Stöckert Centrifugal Pump Console (SCPC) by Sorin Group) were employed in our patients. Implantation was performed either in the medical intensive care unit or in the catheterization laboratory by a specialized ECLS team consisting of two cardiologists, a perfusionist, and a nurse. In case of elective implantation, patients were lightly analgosedated using midazolam (1–3 mg i.v.) and a continuous administration of remifentanil (averaging 0.05 to 0.1 *μ*g/kg/min). The ECLS system was primed with crystalloid fluid and 5000 IU of unfractionated heparin. Cannulation of the femoral vein and artery was performed under sonographic control using Seldinger technique. The diameter of the femoral artery was measured by ultrasound prior to implantation in elective patients. An arterial cannula of a size not exceeding two-thirds of the arterial diameter was chosen in order to avoid limb ischemia. Typically, an arterial cannula (MAQUET Medical, length 20 cm) with a diameter of 15 Fr. (40%) or 17 Fr. (60%) was used. For venous drainage, a venous cannula (MAQUET Medical, length 38 cm) with a diameter of 19 Fr. (26.7%) or 21 Fr. (73.3%) was employed.

### 2.3. PCI

An 8 Fr. femoral approach was used in all patients to allow for an all option approach. During catheterization, unfractionated heparin aiming at an ACT (activated clotting time) of 200 to 250 seconds was administered. Drug eluting stents were implanted in all and a guideline conform dual antiplatelet therapy was initiated in all patients.

### 2.4. ECLS Management during PCI

After implantation of the ECLS system in elective patients, blood flow was adjusted to 2.0 to 2.5 L/min and a gas flow of 1.5 to 2.0 L/min using 50–75% O_2_ was employed. In case of cardiogenic shock, ECLS was adjusted to hemodynamically stabilize the patient. Unfractionated heparin was administered aiming at an ACT between 200 and 250 seconds during PCI and later at a partial thromboplastin time of 50 to 60 seconds. Invasive blood pressure and oxygen saturation were continuously monitored by right radial artery and finger clip approach. Patients were lightly analgosedated continuing using remifentanil. Beside the interventional team, a perfusionist and one physician of the ECLS team were present in the catheterization laboratory during PCI.

### 2.5. ECLS Explantation

The femoral artery ECLS cannula was removed and access site was compressed using the FemoStop Gold Compression Assist device (St. Jude Medical) for at least one hour as previously described [21] followed by a compression bandage for 24 hours. Venous ECLS cannula was simultaneously removed and compressed using the Safeguard device (MAQUET Medical).

### 2.6. Ethics Approval

Ethics approval was obtained by the Ethics Committee of the University of Freiburg (Approval number 151/14).

## 3. Results

### 3.1. Survival and Procedural Risks

Unprotected left main PCI could be successfully performed in all patients. Therefore, no conversion to coronary artery bypass surgery was necessary. No deaths or thromboembolic events occurred in our cohort within the hospital stay (Table 2). The neurological outcome of all patients was excellent with no clinical evidence for a procedural stroke.

### 3.2. Long Term Survival

Mean ambulant follow-up time was 12.2 ± 5.4 months. Within this time, two out of sixteen patients died, one after 9 months of ischemic biliary disease and one after 20 months from myocardial infarction. The localization of the myocardial infarction could not be determined. One patient presented with an in-stent restenosis 16.6 months after left main intervention, which could be managed by PCI without ECLS backup.

### 3.3. ECLS during PCI

ECLS implantation could be successfully performed in all 16 patients using a femoral approach even in patients with reported peripheral artery disease. Six of the seven patients presenting with acute myocardial infarction developed congestive heart failure or cardiogenic shock during PCI and were ECLS dependent for circulatory support. Among the elective coronary interventions, two out of nine patients were ECLS dependent during PCI. Left main PCI was not hampered by ECLS in any of our patients.

ECLS could be explanted in 75% of patients within 12 hours after PCI and in 87.5% of patients within 24 hours. Concerning the elective group, ECLS explantation within 12 hours was possible in all patients. The longest time on ECLS was 96 hours in a patient presenting with STEMI. 31.3% of all patients were intubated during the PCI. All elective patients tolerated ECLS and left main PCI in light analgosedation and none had to be intubated during or following PCI.

### 3.4. Complications

No death, thromboembolic, or ischemic event, including critical limb ischemia after ECLS implantation, occurred within the hospital stay for the index event (Table 3).

Major complications were defined as complications requiring any further medical attention or checkups including laboratory tests. Specifically, three patients developed access complications requiring surgical intervention, all of which had a 17 Fr. arterial cannula. Specifically, one patient suffering from active Takayasu arteritis presented with prolonged bleeding from the arterial access site, which required surgical intervention. One patient developed an occlusion of the superficial femoral artery which was treated by thromboendarterectomy. In one patient a false aneurysm of the femoral artery developed, which was closed surgically.

A total of four patients required packed red blood cells after ECLS implantation. Specifically, two of the three patients requiring surgery had to be transfused with two units of packed red blood cells each, due to access site bleeding after ECLS explantation. One patient dropped to hemoglobin of 7.6 g/dL after implantation of the ECLS (probably due to dilution or blood loss during PCI as no bleeding site was detected) and was transfused with two units of packed red blood cells. One patient developed a prolonged access site bleeding, with required three units of packed red blood cells but could be managed conservatively.

Three patients developed minor complications including small hematoma or minor bleedings. One patient developed a paradox reaction on midazolam [22].

## 4. Discussion

Unprotected left main PCI could be successfully performed in all patients. ECLS was implanted in all patients using the Seldinger technique. Two out of nine patients in the elective group actually needed the ECLS support during coronary intervention whereas six out of seven patients with an acute myocardial infarction were ECLS dependent during the procedure. No patient died during the index hospital stay. Therefore, ECLS implantation as backup system for left main PCI appears to be effective. Considering the high mortality of myocardial infarction with left main artery culprit lesion, ECLS might offer a promising tool avoiding cardiogenic shock in patients with acute coronary syndromes and left main culprit lesion. Several other devices have been tested as backup system for high-risk PCI demonstrating mixed results. The recently published PROTECT II trial shows an improved event-free survival in the Impella 2.5 group when compared to IABP [14]. Which support system will offer the best safety and efficiency profile of each individual patient still has to be determined.

The high numbers of complications we have to report (need for surgical intervention in 18.8% and bleeding requiring transfusion in 25.0% of all patients) however will have to be reduced through further refinement of the approach. In a recent multicenter IABP trial, access site complications requiring interventions were encountered significantly less frequently (4.3%); bleedings however were also common (severe 3.3%, moderate 17.3%) [9]. A single-center study of ECLS in cardiogenic shock reported bleeding complications in 17.4% and need for surgical intervention in 8.7% of all patients [23] while registry data for ECLS in cardiopulmonary resuscitation report complications directly related to ECLS implantation in every third patient [16]. Concerning blood transfusions after device implantation, a recent meta-analysis reported a transfusion in 32 to 38% of all patients after IABP or Impella 2.5 implantation, respectively [24].

In a recent publication on ECLS cannula removal, an open surgical approach was suggested in all patients [25]. In our patients, however, surgical intervention was necessary in less than 20% of all patients and in 0% of patients after 15 Fr. cannulation. In order to reduce complications, employing arterial cannulae with a smaller diameter (15 Fr. instead of 17 Fr.) appears to be a first step since in our patients all complications requiring surgery occurred after placement of a 17 Fr. cannula.

## 5. Conclusion

Unprotected left main PCI could be safely and effectively performed after ECLS implantation as backup in acute coronary syndromes in our patient collective. This holds true also for patients without the need for general anesthesia. The high number of access site complications however needs to be taken into account when considering the use of ECLS. Particularly for elective patients, a 15 Fr. arterial cannula should be employed.

## Figures and Tables

**Table 1:** Patient characteristics.

Demographics	
Age, mean	68.3 ± 10.0 years
Men	13 (81.3%)
Medical history	
Diabetes mellitus	8 (50.0%)
Hypertension	14 (87.5%)
Known KHK	16 (100.0%)
Prior stroke	3 (18.8%)
Peripheral artery disease	2 (12.5%)
Creatinine	1.2 ± 0.4 mg/dL
eGFR [MDRD]	74.0 ± 38.4 mL/min/1.72 m^2^
Presentation	
STEMI	1 (6.3%)
NSTEMI	6 (37.5%)
Unstable angina	9 (56.3%)
Post-CPR^*^	2 (12.5%)
Congestive heart failure	5 (31.3%)
ECLS indication	
Cariogenic shock	6 (37.5%)
Acute myocardial infarction	6 (37.5%)
Unstable angina	0 (0.0%)
Prophylactic (high-risk profile)	10 (62.5%)
Acute myocardial infarction	1 (6.3%)
Unstable angina	9 (56.3%)
ECLS management	
Intubation during PCI	5 (31.3%)
MAQUET CARDIOHELP	6 (37.5%)
Stöckert SCPC^**^	10 (62.5%)
ECLS < 12 h	12 (75.0%)

Table highlighting patients' characteristics of all patients undergoing LMCA PCI with ECLS backup. ^*^Cardiopulmonary resuscitation (CPR) prior to hospital admission. ^**^Stöckert Centrifugal Pump Console (SCPC).

**Table 2:** Outcomes.

Mean follow-up	12.2 ± 5.4 months
Successful left main PCI	16 (100.0%)
In-stent restenosis	1 (6.3%)
Cardiac death	1 (6.3%)
Any death within follow-up	2 (12.5%)

Outcome of high-risk patients undergoing unprotected left main PCI.

**Table 3:** Procedural complications.

No complications	8 (50.0%)
Any complications	8 (50.0%)
Any major complication	5 (31.3%)
Any minor complication	3 (18.8%)
Access site bleeding requiring transfusion	4 (25.0%)
Access site complication requiring surgery	3 (18.8%)
Paradox reaction on sedation	1 (6.3%)
Thromboembolic or ischemic events	0 (0.0%)
Death	0 (0.0%)

Table showing procedural complications encountered in patients undergoing LMCA PCI with ECLS backup. Major complications were defined as complications requiring any further medical attention or checkups including laboratory tests.

## Limitations

Data presented derived from a single-center registry without a control group. Patients were preselected and represent a heterogeneous patient group. Extrapolating findings to a general population is therefore impossible. A large, multicenter randomized trial is required in order to evaluate the net clinical benefit of ECLS implantation as backup system for unprotected left main PCI.
